# Supplementary material for: QTL Mapping of Yield-Related Traits in Tetraploid Wheat Based on Wheat55K SNP Array
Source: Plants (Basel). 2024 May 7;13(10):1285. doi: 10.3390/plants13101285 (PMC11125051; doi:10.3390/plants13101285)
Supplement: Supplementary file 1 [file plants-13-01285-s001.zip › Figure Supplement 1.pdf]

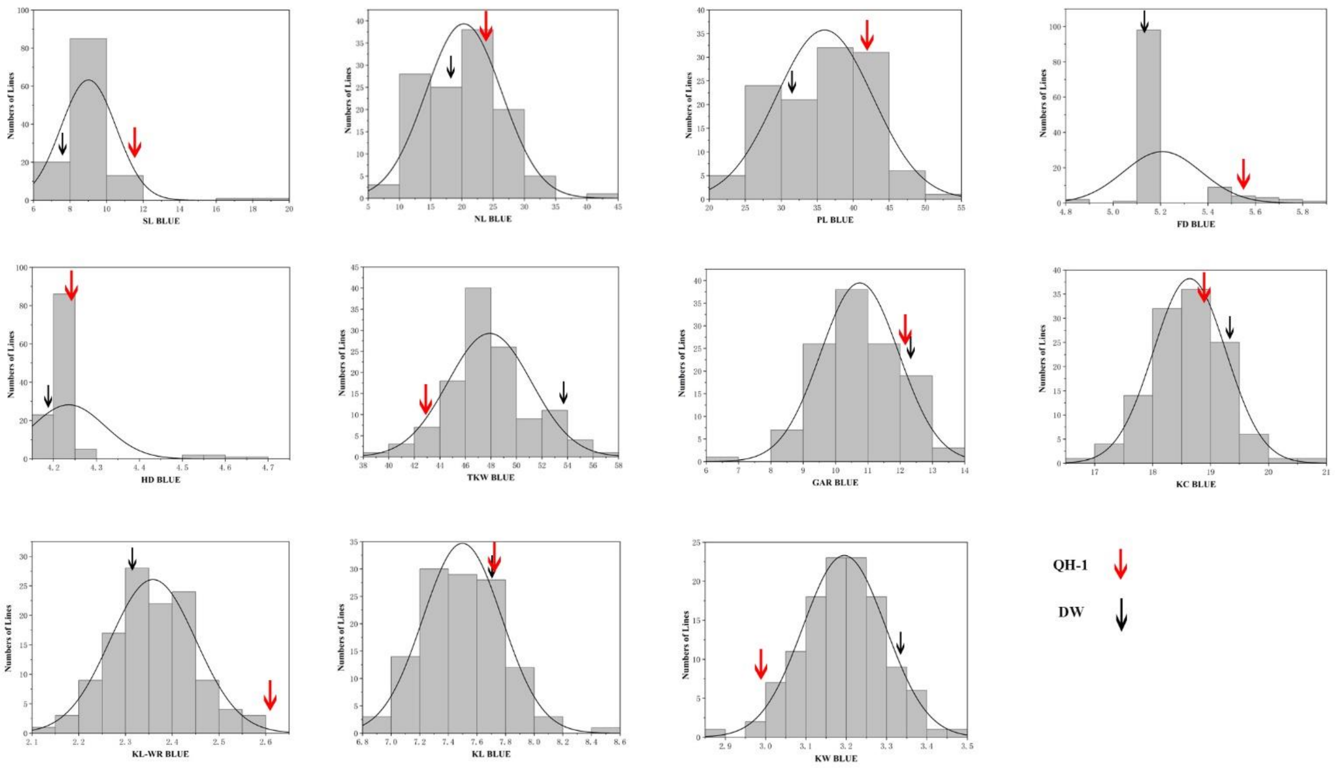

Figure Supplement S1. Frequency distributions for Spike length(SL), neck length(NL), peduncle length(PL), flowering date(FD), heading date(HD), thousand-kernel weight(TKW), Kernel area ratio(KAR), kernel circumference(KC), kernel length(KL), kernel width(KW) and kernel length-width ratio(KL-WR)based on BLUE.
