# Supplementary material for: QTL Mapping of Yield-Related Traits in Tetraploid Wheat Based on Wheat55K SNP Array
Source: Plants (Basel). 2024 May 7;13(10):1285. doi: 10.3390/plants13101285 (PMC11125051; doi:10.3390/plants13101285)
Supplement: Supplementary file 1 [file plants-13-01285-s001.zip › Figure Supplement 2.pdf]

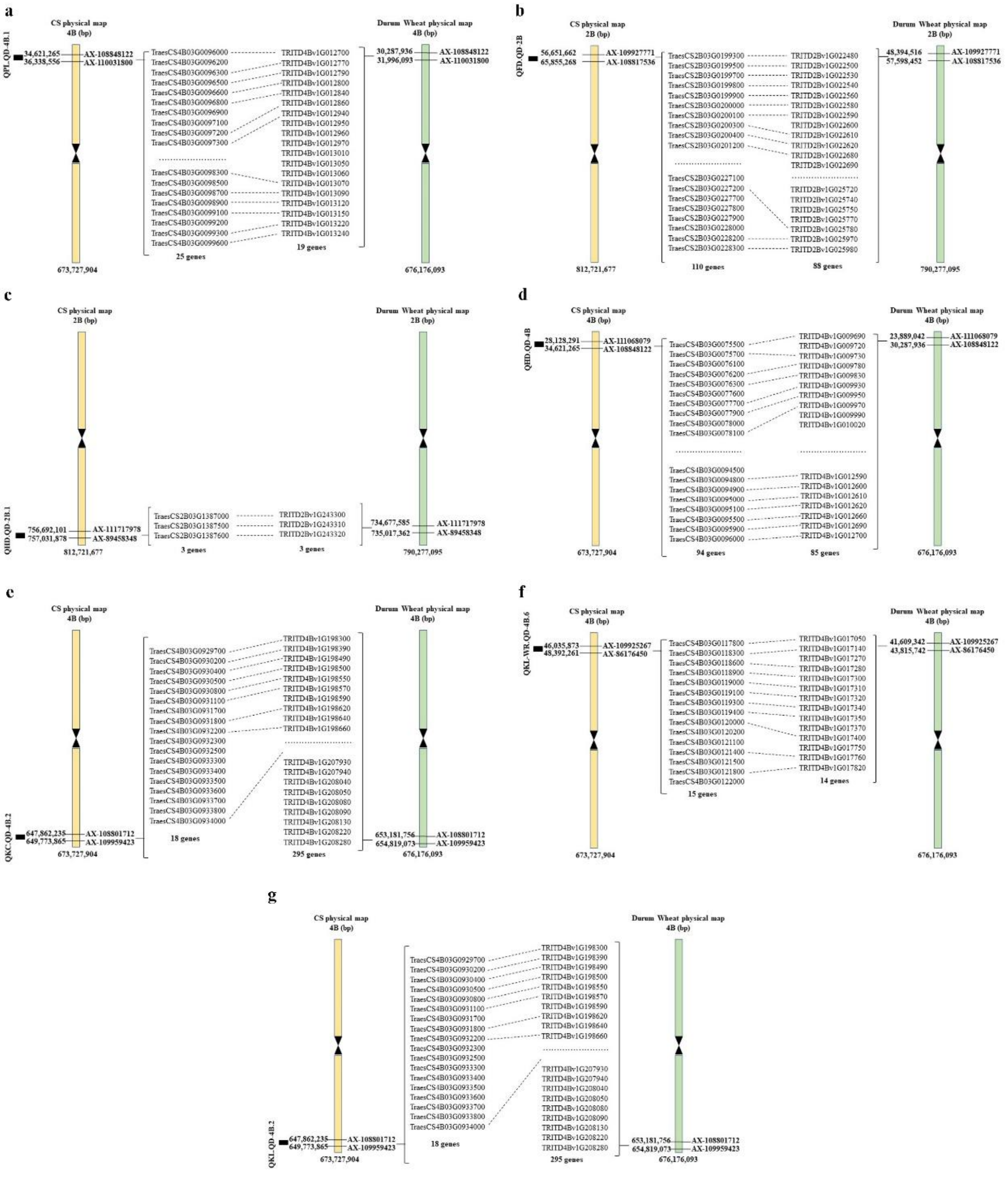


Figure Supplement S2. Physical maps of the *QPL.QD-4B.1*(a), *QFD.QD-2B*(b), *QHD.QD-2B.1*(c), *QHD.QD-4B*(d),, *QKC.QD-4B.2*(e),, *QKL-WR.QD-4B.6*(f), and *QKL.QD-4B.2*(g) on ‘CS’ and Durum Wheat; dotted lines indicate homologous genes
